# Homology recognition without double-stranded DNA-strand separation in D-loop formation by RecA

**DOI:** 10.1093/nar/gkad1260

**Published:** 2024-01-12

**Authors:** Takehiko Shibata, Shukuko Ikawa, Wakana Iwasaki, Hiroyuki Sasanuma, Hisao Masai, Kouji Hirota

**Affiliations:** Department of Chemistry, Graduate School of Science, Tokyo Metropolitan University, 1-1 Minami Ohsawa, Hachioji, Tokyo 192-0397, Japan; Genome Dynamics Project, Department of Basic Medical Sciences, Tokyo Metropolitan Institute of Medical Science, 2-1-6 Kamikitazawa, Setagaya-ku, Tokyo 156-8506, Japan; Cellular & Molecular Biology Laboratory, RIKEN, Wako-shi, Saitama 351-0198, Japan; Cellular & Molecular Biology Laboratory, RIKEN, Wako-shi, Saitama 351-0198, Japan; Laboratory for Translation Structural Biology, RIKEN Center for Biosystems Dynamics Research, 1-7-22 Suehiro-cho, Tsurumi-ku, Yokohama, Kanagawa 230-0045, Japan; Genome Dynamics Project, Department of Basic Medical Sciences, Tokyo Metropolitan Institute of Medical Science, 2-1-6 Kamikitazawa, Setagaya-ku, Tokyo 156-8506, Japan; Genome Dynamics Project, Department of Basic Medical Sciences, Tokyo Metropolitan Institute of Medical Science, 2-1-6 Kamikitazawa, Setagaya-ku, Tokyo 156-8506, Japan; Department of Chemistry, Graduate School of Science, Tokyo Metropolitan University, 1-1 Minami Ohsawa, Hachioji, Tokyo 192-0397, Japan

## Abstract

RecA protein and RecA/Rad51 orthologues are required for homologous recombination and DNA repair in all living creatures. RecA/Rad51 catalyzes formation of the D-loop, an obligatory recombination intermediate, through an ATP-dependent reaction consisting of two phases: homology recognition between double-stranded (ds)DNA and single-stranded (ss)DNA to form a hybrid-duplex core of 6–8 base pairs and subsequent hybrid-duplex/D-loop processing. How dsDNA recognizes homologous ssDNA is controversial. The aromatic residue at the tip of the β-hairpin loop (L2) was shown to stabilize dsDNA-strand separation. We tested a model in which dsDNA strands were separated by the aromatic residue before homology recognition and found that the aromatic residue was not essential to homology recognition, but was required for D-loop processing. Contrary to the model, we found that the double helix was not unwound even a single turn during search for sequence homology, but rather was unwound only after the homologous sequence was recognized. These results suggest that dsDNA recognizes its homologous ssDNA before strand separation. The search for homologous sequence with homologous ssDNA without dsDNA-strand separation does not generate stress within the dsDNA; this would be an advantage for dsDNA to express homology-dependent functions *in vivo* and also *in vitro*.

## Graphical Abstract

**Graphical Abstract:**
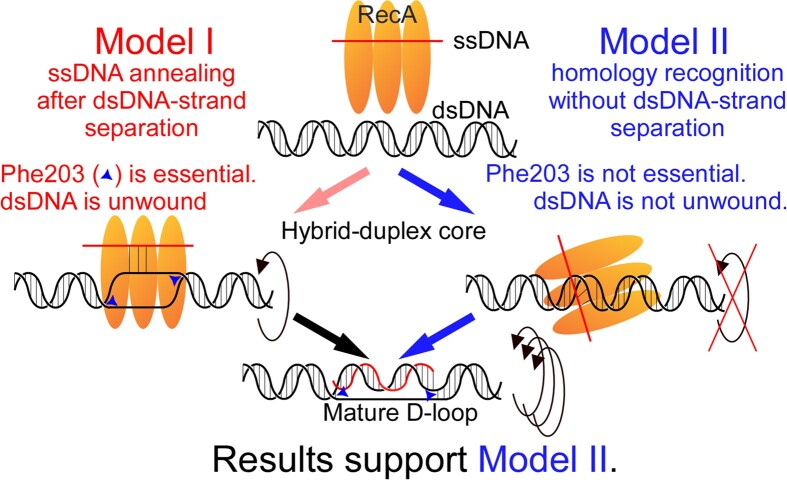

## Introduction

Homologous recombination is a general genetic phenomenon observed in virtually all living creatures. Homologous recombination repair of DNA damage is required for stable genome maintenance (see ref. 1 for a review) and meiotic recombination for gamete formation in sexual reproduction (see ref. 2 for a review). All homologous recombination of genomic DNA requires RecA or a RecA/Rad51 orthologue, except for some viruses (3) and mitochondria (4); i.e. RecA in eubacteria (5–7), Rad51 in eukaryotes (8,9), and meiosis-specific Dmc1 (10).

Holliday described the mechanism of meiotic homologous recombination as a processing of hybrid-duplex products (‘hybrid region’) formed symmetrically on both parental dsDNA (11), but he did not explain how hybrid-duplex products were formed. Homologous recombination is initiated by single-strand-gap formation (12) or by a double-strand break followed by resection to generate single-stranded (ss)DNA tails (13, see ref. 14 for review). RecA (15–17), Rad51 (18) or Dmc1 (19) then pairs the ssDNA region (tail or gap) with an internal homologous sequence of dsDNA in the presence of ATP, forming a D-loop. D-loops are obligatory recombination intermediates that consist of a hybrid-duplex product (or heteroduplex product: (20)) of the ssDNA and the complementary strand of the internal homologous sequence of the partner dsDNA and a loop made of the displaced strand of the dsDNA (15,16,21). The question is, how does RecA/Rad51 mediate the recognition of homology between ssDNA and an internal sequence dsDNA to form a hybrid-duplex product?

The biochemical process of D-loop formation from homologous ssDNA and dsDNA by RecA and RecA/Rad51 orthologues is best understood through the example of *Escherichia coli* RecA (see refs. 22–25 for review). D-loop formation consists of two reaction phases: ATP-dependent but ATP hydrolysis-independent homology recognition to form a hybrid-duplex core (6–8 bp: 26,27) (‘homologous pairing’) and the extension of the core in 3 base-steps up to less than 14 base pairs (28), and ATP hydrolysis-dependent hybrid-duplex/D-loop processing. RecA protomers first bind cooperatively, in a head-to-tail fashion, to ssDNA, and form a RecA-ssDNA spiral filament. The binding of ATP to RecA elongates and activates the filament for dsDNA binding and ATP hydrolysis. The dsDNA then binds to the activated RecA spiral filament, independent of sequence homology, and homologous sequences between dsDNA and ssDNA are recognized within the RecA-ATP-ssDNA-dsDNA complex (26,29–31). It was shown that two adjacent RecA protomers in head-to-tail contact are sufficient to form the hybrid-duplex core (32). Thus, the filament structure is not an absolute requirement for homology recognition to form the hybrid-duplex core (32). Playing a supportive role in homology recognition, filament formation provides a number of sites on each of the filaments to allow the dsDNA to attempt hybrid-duplex-core formation in a three-dimensional space, resulting in the acceleration of D-loop formation (33,34).

Hybrid-duplex product/D-loop processing is observed in one of two ways: hybrid-duplex extension of up to thousands of base pairs (called ‘branch migration’) or D-loop dissociation. Hybrid-duplex extension is observed when a hybrid-duplex product is formed at one end of the linear dsDNA with ssDNA without end (i.e. circular ssDNA) (35,36), and D-loop dissociation (37–39) is observed when the dsDNA is circular dsDNA without strand disruption, called closed circular dsDNA (cc-dsDNA) with ssDNA with a terminus, as in the case of homologous recombination *in vivo* (13,40,41). D-loop dissociation is a result of unidirectional D-loop migration to the 3′ end of the homologous ssDNA by RecA with ATP hydrolysis (37). Most D-loop formation likely starts at a sequence at some distance away from the end of the ssDNA (42); thus the size of the D-loop is limited by topology until an end of the D-loop meets the end of ssDNA (43). D-loop dissociation has the following features: (i) it depends on the filament formation of RecA (44,45), (ii) When dsDNA has a break(s), D-loop dissociation was not observed (46), (iii) it is an active reaction dependent on ATP hydrolysis (does not occur in the presence of ATPγS: 32), (iv) it is more sensitive to inhibition by ADP than is D-loop formation (38), and (v) it exhibits a net polarity of 5′ to 3′ relative to the ssDNA paired with the homologous dsDNA (39).

**Figure 1.**
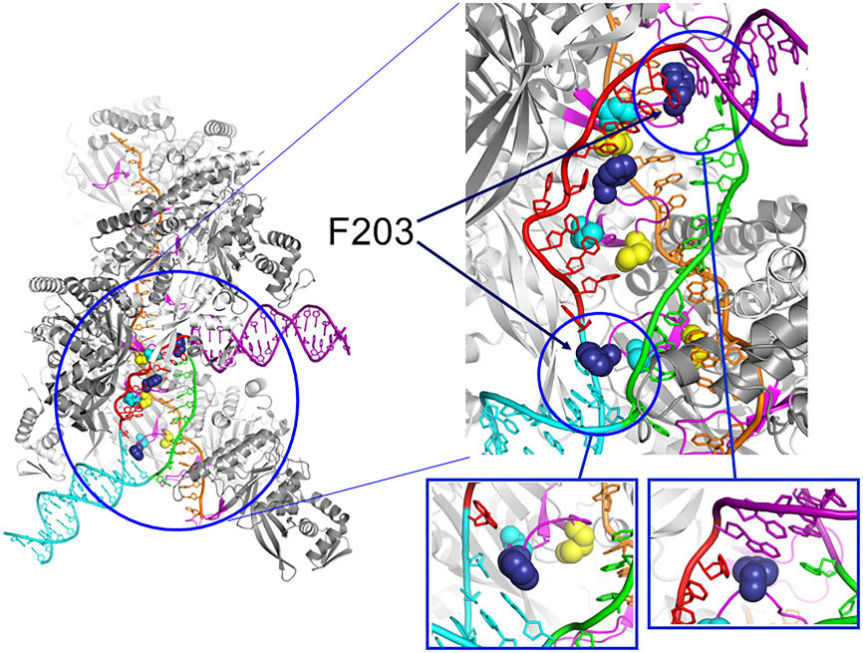
Molecular structures around the L2 loop and a D-loop in the RecA spiral filament-dsDNA complex. dsDNA binds to and enters into the RecA-ATP-ssDNA spiral filament through a ‘gateway,’ starting in the C-terminal domain of a RecA protomer, and is lead to the L2 loop region where the dsDNA interacts with ssDNA in the filament to detect sequence homology (79–81). At each end of a D-loop, Phe203 (indicated by arrows) stacks with the last base pairs of the dsDNA flanking the D-loop and prevents pairing of the first bases in the separated strands; thus Phe203 stabilizes strand separation of the parental dsDNA (42). This figure was created with PyMOL (Schrödinger LLC) using the cryo-EM structure of RecA-D-loop complex (PDB code: 7jy9). Colors: orange, ssDNA; magenta, L2 loop (residues 195-209); side chains: yellow, Met 197; blue, Phe203; cyan, Asn205.

Two competing and mutually exclusive models have been proposed by researchers to explain the molecular mechanism of homology recognition: ‘ssDNA annealing after dsDNA-strand separation’ and ‘homology recognition without dsDNA-strand separation’. The homology recognition without dsDNA-strand separation model is supported by biochemical (47), NMR (48), and thermodynamic (49) studies as well as by single-molecule analysis (50). However, ssDNA annealing after dsDNA-strand separation appears more straightforward. A recent study using cryo-electron microscopy (cryo-EM) at atomic resolution revealed the detailed structure of the *E. coli* RecA–ATPγS–ssDNA–dsDNA complex containing a D-loop. The authors of this study make the following points in support of the ssDNA annealing after dsDNA-strand separation model (42). First, in complexes containing heterologous ssDNA and dsDNA, the two strands of the dsDNA appear separated in an internal region, as judged by the absence of reconstructed cryo-EM maps for the complementary strand in the solved structures. Second, in structures containing a D-loop, at both ends of the D-loop region of dsDNA where one of the strands forms a hybrid-duplex product with the ssDNA in the RecA-spiral filament and the other strand is in single-strand, phenylalanine at position 203 (Phe203) stacks with the last base pairs of the flanking double-stranded regions and prevents the pairing of the first-opened base pairs. Thus, Phe203 stabilizes strand separation in this region of the dsDNA (42: Figure 1). Note that the amino-acid-residue number of the RecA protein starts from Ala on the second codon: The N-terminal residue was identified as ‘Ala’ in the first publication on N-terminal-amino-acid sequencing along with the DNA sequence of the *recA* gene (7).

Phe203 is the conserved aromatic residue at the tip of the β-hairpin loop called the L2 loop (42,51) among various eubacterial RecA (52). The stacking of aromatic residue at a tip of the β-hairpin, with the last base pairs has been widely observed at the opening where the strands of dsDNA are separated. For example: the Phe residue on the β-hairpin that plays a role in strand separation by a superfamily-2 DNA helicase (53), and the tryptophan residue on the β-hairpin of a leading strand polymerase that functions in dsDNA-strand separation at the replication fork by the phage T7 replisome, a complex of the leading-strand DNA polymerase and fused DNA helicase-DNA primases (54). These observations suggest that Phe203 at the tip of the L2 loop plays a role in dsDNA-strand opening to allow the single strands thus formed to anneal with ssDNA in RecA-mediated D-loop formation.

In this study, we tested the straightforward model, ssDNA annealing after dsDNA-strand separation, by analyzing the effects of the alanine (Ala, A) replacement of Phe203 (F203A). We found, contrary to the prediction of the ssDNA annealing after dsDNA-strand separation model, that a mutant recA protein lacking the aromatic residue (recA F203A) can promote homology recognition and hybrid-duplex product-formation. This suggested homology recognition without dsDNA-strand separation. We tested this suggestion using a sensitive dsDNA unwinding assay, and found that during search for a homologous sequence between dsDNA and ssDNA in the RecA-ATP-ssDNA-dsDNA complex, the dsDNA of approximately 3000 bp was not unwound by even a single turn of the double helix, and that the dsDNA was unwound only after a homologous sequence with ssDNA was recognized. These results support the homology recognition without dsDNA-strand separation model.

## Materials and methods

### DNA

#### Plasmid supercoiled cc-dsDNA (Form I)

Plasmid closed circular double stranded (cc-dsDNA) isolated from cells has negative (right-handed) supercoils. Note that alkaline and other treatments causing denaturation have to be avoided during plasmid-DNA preparation. Any treatment to denature cc-dsDNA with negative supercoils leaves a non-B-form structure after renaturation, since negative supercoils stabilize various non-B-form structures, such as ssDNA regions, the Z-DNA, the H-form three stranded DNA and G-quadruplex. RecA preferentially interacts with non-B-form structures (55,56), and the interactions could affect RecA-mediated D-loop formation. pBlueScript II SK(−) plasmid dsDNA (2961 bp) and pKF18 plasmid dsDNA (2203 bp) were purified from supernatant obtained by ultracentrifugation of gently lysed cells containing the plasmid, followed by phenol extraction, alcohol precipitation and sucrose-density-gradient centrifugation to separate plasmid cc-dsDNA from plasmid DNA of nicked circular or linear form, and from fragmented cellular DNA, as described previously (57). When sucrose-gradient centrifugation is not available, plasmid Form I DNA can be separated from other forms of plasmid DNA, cellular DNA and RNA by Sephacryl S-1000 gel filtration (GE Healthcare: 58).

#### Nicked circular dsDNA (circular dsDNA with a nick [single-strand break] at a specific site): substrate Form II DNA

Nicked circular dsDNA (substrate Form II DNA) was prepared by treatment of plasmid Form I DNA (100 μM in nucleotides) with restriction endonuclease *Alu*I (Takara Bio, Shiga, Japan) in the presence of ethidium-bromide (152 μM, 60 μg/ml) in an ‘M’ buffer (Takara Bio) for 60 min at 37°C. Ethidium-bromide allows the cutting of one strand at only one cutting site and prevents cleavage of the other strand at that site and of strands at other sites. Under these conditions, linear dsDNA (Form III) is unavoidably produced. The amount of *Alu*I has to be determined (0.02 U/μl, for example) for each enzyme preparation to obtain a complete cut of Form I and to minimize the production of Form III. We obtained a nicked circular dsDNA preparation containing 84–89% nicked circular form, 10–16% linear form and 1% uncut Form I, from a Form I preparation containing 5% nicked circular form. Ethidium-bromide has to be removed as completely as possible in DNA preparation, since it affects the interaction of RecA and DNA. Ethidium-bromide was removed by extraction with phenol-chloroform-isoamyl alcohol (25:24:1 mixture) adjusted to pH 7.9, followed by alcohol precipitation in the presence of Na acetate (0.3 M) and ethanol (70%) washing. Finally, the DNA was dissolved in Tris–HCl buffer (10 mM; pH 7.5) containing EDTA (0.1 m), and subjected to buffer change and concentration using an Amicon^®^ Ultra-15 10K Centrifugal Filter Unit.

#### ssDNA preparation and 5′ end-labeling with ^32^P

OL2 90-mer ssDNA is defined as: AAATCAATCT AAAGTATATA TGAGTAAACT TGGTCTGACA GTTACCAATG CTTAATCAGT GAGGCACCTA TCTCAGCGAT CTGTCTATTT (5′ to 3′: see ref. 59 about the origin). Km90 90-mer ssDNA is defined as ATCTGATCCT TCAACTCAGC AAAAGTTCGA TTTATTCAAC AAAGCCACGT TGTGTCTCAA AATCTCTGAT GTTACATTGC ACAAGATAAA (5′ to 3′: 32). OL2 and Km90 were purchased from Eurofins Genomics K.K. (Tokyo, Japan). 5′ end-labeling with ^32^P was described previously (32). The M13mp18 and ΦX174 phage circular ssDNA were purchased from Takara Bio (Shiga, Japan) and New England Biolabs Japan (Tokyo, Japan), respectively.

#### Standard reaction mixture

The standard reaction mixture contains Tris–HCl buffer (31 mM; pH 7.5 ± 0.1), MgCl_2_ (13 mM), ATP (1.3 mM), bovine serum albumin (88 μg/ml) and dithiothreitol (1.8 mM) (37). The reaction mixture pH was adjusted to 7.5 in the presence of all components. Note that the pH of Tris–HCl buffer changes according to dilution. The pH of the ATP stock solution should be adjusted to 7.5.

#### Purification of RecA variants

Described previously (59 and its supplemental methods).

#### ATPase assay

The amount of ATP was measured by the luminescence emitted by a luciferase–luciferin reaction. Reaction mixture (21 μl) containing 1.3 mM unlabeled ATP, 20 μM ΦX174 ssDNA and the indicated amount of RecA or recA F203A, was divided into 4.8 μl aliquots in 0.6 ml tubes, each of which was incubated for 10–30 min at 37°C. The ATP hydrolysis was terminated by chilling in ice-water bath. Each aliquot was diluted 1630 folds with H_2_O in 2 steps, and to 50 μl of each diluted aliquot, 10 μl CellTiter-Glo^®^ Reagent (Promega Corporation, USA) was added and mixed well. The luminescence was measured in 96-well black titer plates using PerkinElma 2030 Multilabel Reader ARVO ^®^ X2 (PerkinElmer Life and Analytical Sciences, Turku, Finland), and the initial rates of ATP hydrolysis (mM/min) were obtained. We confirmed that the signal depends linearly on the concentration of ATP between 0.01 and 1 μM in 50 μl samples under these conditions. The ATPase activities of other RecA variants were measured using [α-^32^P]ATP as described previously (59).

#### D-loop assay

Unless otherwise stated, the component concentrations indicated are the final concentrations of the complete reaction mixtures. Under standard conditions, OL2 90-mer [^32^P]ssDNA (0.06 μM in nucleotides; 0.7 nM in molecules) was incubated in a standard reaction mixture with a RecA variant (2.0 μM) and ATP (1.3 mM) for 5–10 min at 37°C to allow the formation of active RecA–ssDNA spiral filaments. D-loop-formation was initiated by the addition of homologous pBlueScript II SK(–) or heterologous pKF18 Form I DNA (plasmid supercoiled cc-dsDNA; 18 μM in nucleotides; 3.0 nM [pBlueScript] or 4.1 nM [pKF18] in molecules). Incubation was continued in the reaction mixture (41 μl), and at the indicated time, 4.5 μl aliquots were withdrawn. The reaction was terminated and proteins were removed. Reaction products were analyzed by gel electrophoresis, and the amounts of ^32^P in the D-loops and in unreacted ssDNA were measured by use of Imaging Plates and a bio-imaging analyzer. The details of the D-loop assay were described previously (59). We added an ATP-regenerating system containing phosphocreatine (4.8 mM) and creatine phosphokinase (7.8 units/ml; Sigma Chemical Co.) in the earlier experiments, but removed the system in the later ones, since we noticed that under the present conditions with the small amount of ssDNA, the addition of the ATP-regenerating system showed no effects on the D-loop formation and following dissociation.

#### Unwinding assay

The component concentrations indicated are the final concentrations of the complete reaction mixtures, just before the addition of T4 DNA ligase. Under standard conditions, homologous (OL2) or heterologous (Km90) 90-mer ssDNA (1.1 μM in nucleotides; 12 nM in molecules) was incubated with RecA^+^ (4.6 μM) in the presence of ATP (4 mM) for 5 min at 37°C; the reaction was initiated by the addition of pBlueScript II SK(–) nicked circular dsDNA (substrate Form II DNA, 10 μM in nucleotides; 1.7 nM in molecules). Incubation was continued in a reaction mixture (11 μl) for 40 min at 37°C. T4 DNA ligase was then added at 1.6 U/μl with ATP at 0.17 mM (final concentration in addition to the ATP previously added) as a 1.2 μl solution, followed by incubation for 2 min at 37°C. To terminate the reaction, the reaction mixture was chilled on ice, and a phenol-chloroform-isoamyl alcohol (60 μl; 25:24:1) mixture at pH 7.9 was added, mixed, and centrifuged. 10 μl of the upper aqueous phase was recovered. 1 μl 10-fold concentrated loading buffer (50% [w/v] glycerol, 0.05% [w/v] bromophenol blue) was added and the mixture was loaded into wells (4 mm w × 1 mm h × 0.35 mm d) of agarose gel (1.4% w/v; 6 cm h × 11 or 5 cm w × 0.4 cm d). The gel was subjected to two-dimensional electrophoresis in TAE buffer (Tris acetate buffer [40 mM], pH 8.0, EDTA [1 mM]: 60) in the presence of ethidium-bromide (15 nM in the first dimension and 150 nM in the second dimension) at room temperature. TAE buffer containing ethidium-bromide should be prepared for each electrophoresis. The agarose gel (isolated from gel-tray) remained with gentle shaking in the TAE buffer containing the indicated concentration of ethidium-bromide to equilibrate the ethidium-bromide concentration for >45 min before each electrophoresis. After electrophoresis, the gel was stained with ethidium-bromide (1.3 μM; 0.5 μg/ml) for 30 min, and washed with an excess amount of H_2_O for more than 3 h at room temperature. Gel images were taken under UV irradiation using the FAS-IV imaging system with a CCD camera (Nippon Genetics Ltd). Ethidium bromide concentration was determined by absorbance at 480 nm and *E*^1M^_480nm_ = 5450 (61).

## Results

### Construction of Ala-replacement mutants of Phe203 and other selected residues in the L2 loop

Starting with the pKK223-3-RecA plasmid, in which the wild-type *recA* (*RecA*^+^) is regulated under the Tac promoter, and pET3a-RecA plasmid in which *RecA^+^* is regulated under T7 promoter, we replaced Phe203 on the L2 loop of RecA with Ala to obtain *recA F203A* expression vectors. We also constructed a series of *recA* variants with an amino-acid replacement on the L2 loop using the pKK223-3-RecA plasmid. Glutamine at 194 (Gln194) and arginine at 196 (Arg196) on the L2 loop are involved in the hydrogen-bond network for ATP binding, ssDNA binding, and the orientation of adjacent RecA protomers to elongate the RecA-ssDNA filament (51). We replaced Gln194 and Arg196 with Ala to obtain *recA Q194A* and *recA R196A*, respectively, as negative controls. In addition, we selected amino-acid residues on the L2 loop, methionine at 197 (Met197) and asparagine at 205 (Asn205), which interact with a strand of dsDNA or ssDNA (42), and constructed Ala-replacement mutants *recA M197A* and *recA N205A*. All of these recA variants were expressed by IPTG-induction in *E. coli* host cells with *recA*-deletion mutation (MV1184 for pKK223-3-RecA variants, and a *ΔrecA* derivative of BL21 (DE3) containing pLysS plasmid for pET3a-RecA variants), the cell-free lysates were prepared, and purified by a series of column chromatography. Note that we have never attached a tag for affinity-purification to the RecA variants to avoid unexpected effects.

**Figure 2.**
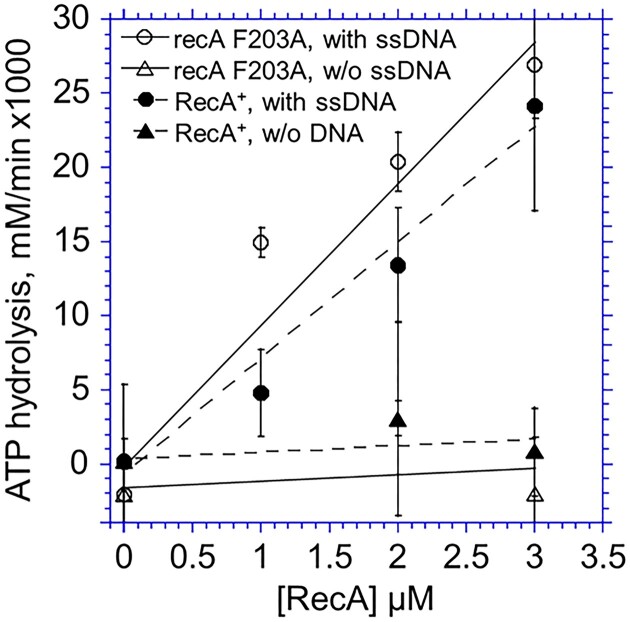
ssDNA-dependent ATP hydrolysis by recA F203A and RecA^+^. ATP (1.3 mM) was incubated with phiX174 phage ssDNA (20 μM) and recA F203A or RecA^+^ at the indicated amounts for 3–4 selected periods between 10–30 min at 37°C. After the reaction was terminated, the amount of ATP was measured by the luminescence emitted by a luciferase-luciferin reaction, and the initial hydrolysis rates were calculated. *N* = 4–8 for RecA^+^ and 2–5 for recA F203A.

RecA has an ATP-hydrolyzing activity dependent on ssDNA (62–64). We analyzed the ability of ATP hydrolysis in the presence of ssDNA. recA F203A has the same (or slightly higher) activity level as the RecA^+^ (Figure 2). Both recA Q194A and recA R196A are completely defective (Supplementary Figure S1). Other Ala-replacement mutants showed the ssDNA-dependent ATP-hydrolyzing activity (Supplementary Figure S1). These results indicate that Phe203, Asn205, and Met197 are not required for ssDNA-dependent ATPase activity of RecA. Since the ATP-hydrolysis requires a structural transition of the RecA–ssDNA spiral filament induced by the binding of ATP and ssDNA (51), it is unlikely that these three amino-acid residues are involved in the structural transition to the active filament.

### Mutant recA F203A promotes D-loop formation

The ability of the RecA variants to form D-loops was assayed by a D-loop assay using OL2 90-mer [^32^P]ssDNA and homologous (pBlueScript II SK(–)) or heterologous (pKF18) plasmid cc-dsDNA, with negative (right-handed) supercoils, Form I. Note that alkaline treatment has to be avoided during plasmid DNA preparations for D-loop assay, as described in Materials and methods.

**Figure 3.**
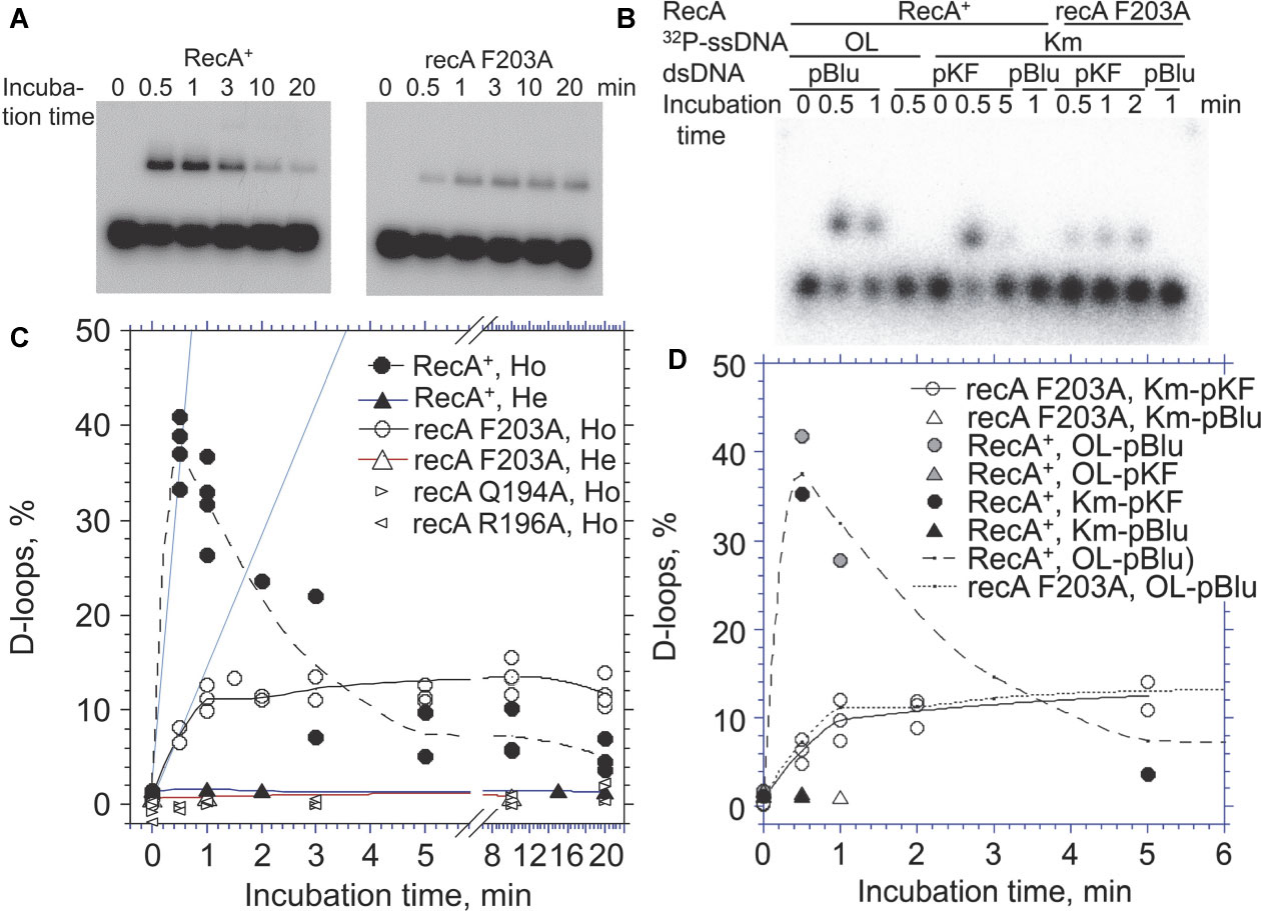
D-loop formation and defective D-loop processing by recA F203A. (**A**, **B**) D-loop formation and dissociation by RecA^+^ and recA F203A shown by electrophoretic profile (A, X-ray film) and the quantitative representation (B). OL2 90-mer [^32^P]ssDNA (0.06 μM in nucleotides) were incubated with RecA variant (2.0 μM) in the presence of ATP (1.3 mM) and with or without an ATP-regenerating system for 5 min at 37°C, and D-loop formation was initiated by the addition of homologous (pBlueScript II SK(−), Ho) or heterologous (pKF18, He) plasmid cc-dsDNA (Form I DNA, 18 μM in nucleotides), and incubated for the indicated time. After removal of the proteins, the DNA products were analyzed by agarose-gel electrophoresis. The radio activity in the gel was measured by use of Imaging Plates and a bio-imaging analyzer. All data from four independent experiments, each with RecA+ and recA F203A, were plotted in a panel. (**C**, **D**) D-loop formation promoted by recA F203A is dependent on the homologous combination of ssDNA and Form I DNA. Km90 90-mer [^32^P]ssDNA (0.06 μM in nucleotides) and homologous (pKF18) or heterologous (pBlueScript II SK(−)) plasmid cc-dsDNA (Form I DNA, 18 μM in nucleotides) were incubated with RecA variant (2.0 μM) in the presence of ATP (1.3 mM) and without an ATP-regenerating system for the indicated time at 37°C. (C) electrophoretic profile obtained from an Imaging plate: OL, OL2; Km, Km90; pKF, pKF18; pBlu, pBlueScript II SK(−). (D) the quantitative representation. Broken and dotted lines represent the D-loop formation by OL2 and pBlueScript II SK(−) by RecA^+^ and recA F203A, respectively, copied from panel B. All data obtained from three independent experiments were plotted.

The amounts of D-loops formed by RecA are expressed by the fraction (%) of ssDNA that is integrated in the D-loops. This fraction is calculated from the strength of ^32^P signals measured after gel electrophoresis to separate ssDNA integrated in D-loops from unreacted ssDNA (Figure 3A and C). RecA^+^ at 1–8 μM gave the maximum initial rate of D-loop formation and did not significantly change with variation in the amount of RecA (Supplementary Data in ref. 59). In the present study, we added RecA at 2 μM, to minimize the effects of experimental fluctuations in the amounts of RecA tested. In these experiments, we used plasmid cc-dsDNA; thus, in the hybrid-duplex product/D-loop processing phase, RecA^+^ dissociates the D-loops during the prolonged incubation, as described in the Introduction (Figure 3A and B). The replacement of the essential amino-acid residues that mediate the communication of ssDNA-binding, ATP-binding, and RecA-ssDNA filament activation (Q194A and R196A) abolished the D-loop formation (Figure 3B, open right and left pointing triangles, respectively).

It has been suggested that Phe203 at the tip of the L2 loop mediates strand-opening of dsDNA to allow the ss strand to anneal with the ssDNA to form a D-loop (see Introduction). Thus, it was expected that the mutant RecA with Ala replacing Phe203 (recA F203A) would be unable to promote D-loop formation. Contrary to this expectation, although the initial rate of D-loop formation was reduced (1/5 of RecA^+^ compared by the initial rate of the formation), recA F203A promoted D-loop formation (Figure 3A and B, open circles). We confirmed that the D-loop formation by recA F203A is solely dependent of the homologous combination of ssDNA and dsDNA (Form I) like RecA^+^; D-loops were formed by recA F203A in the combination of either OL2 and pBlueScript II SK(−) (Figure 3A and B) or Km90 and pKF18 (Figure 3C and D), but not in the combination of OL2 and pKF19 (Figure 3B) or Km90 and pBlueScript II SK(−) (Figure 3C and D). These results indicate that Phe203 is not essential to D-loop formation.

### Mutant recA F203A is deficient in D-loop processing

Figure 3 shows that recA F203A, unlike RecA^+^, did not reduce the amount of D-loops during prolonged incubation after D-loop formation. Two other RecA mutants with amino-acid replacement in the L2 loop, recA M197A and recA N205A, showed reduced rate in D-loop formation, as with the case of recA F203A; but unlike recA F203A, the D-loops formed by recA M197A and recA N205A decreased during prolonged incubation, as with RecA^+^ (Supplementary Figure S2; open reversed triangles and open diamonds, respectively). This indicates that the D-loop dissociation depends on Phe203, but other DNA-interacting amino acid-residues (Met197 and Asn205) in the L2 loop are not required for the D-loop dissociation. These results show that Phe203 plays a unique role in D-loop processing. As these amino acid-residues are not essential to D-loop formation, we did not investigate the issue further in the present study.

### Sensitive assay for dsDNA unwinding

Phe203 appears to play a role in separation of the dsDNA (42), but it was shown that Phe203 is not essential to D-loop formation, suggesting that the dsDNA-strand separation is not required for D-loop formation. Then, we attempted to set up the conditions for a sensitive unwinding assay to test the mutually exclusive models described in the Introduction: ssDNA annealing after dsDNA-strand separation and homology recognition without dsDNA-strand separation.

If the ssDNA annealing after dsDNA-strand separation model is the correct one, since the hybrid-duplex core formed by RecA is 6–8 base pair or larger (26,27), the dsDNA should be unwound at least 0.6–0.8 turns before each trial for annealing with the ssDNA in RecA-ssDNA spiral filament. To make sure that each dsDNA molecule interacts with multiple ssDNA molecules, we added ssDNA molecules at an excess molar ratio to the dsDNA (7-fold). Note that all ssDNA and dsDNA form an huge network with RecA in the presence of ATP but independent of homology of ssDNA and dsDNA as the intermediate of D-loop formation (33), and that a single dsDNA molecule interacts with a number of sites on a RecA-ssDNA spiral filament at the same time, and *vice versa* (34). A study using single molecule fluorescence imaging tools with high spatiotemporal resolution showed that dsDNA slides along the RecA–ssDNA–spiral filament to search for homologous sequence, and that the average lifetime of the dsDNA bound to the complex is 2.5 s in the presence of ATP and a higher concentration of MgCl_2_ (10 mM) (26), both of which are required for D-loop formation (46). Thus, in a reaction mixture containing heterologous dsDNA and ssDNA, where search for homologous sequence continues without end, most of the dsDNA molecules would be unwound several turns at any given time during the reaction. On the other hand, if the homology recognition without dsDNA-strand separation model is the correct one, the dsDNA molecules would not be unwound even a single turn before the homologous sequences are recognized.

To measure dsDNA unwinding with as high a level of sensitivity as possible, we employed circular dsDNA with a single-strand break (nick) to release all rotational stress causing and antagonizing unwinding and all stress induced by possible unwinding. The nick was introduced at a single specific site that did not overlap with the homologous 90-mer ssDNA, to avoid the possible effects on homology recognition with ssDNA and on ligation caused by the presence of a D-loop (See Materials and Methods). To irreversibly trap the dsDNA unwound by the RecA activity, all nicked circular dsDNA molecules in the reaction mixture were ligated, to convert the nicked circular dsDNA to cc-dsDNA, by adding an excessive amount of T4 DNA ligase in the presence of RecA, ssDNA and ATP. The amount of T4 DNA ligase was sufficient to ligate all nicked circular dsDNA molecules within 2 min, but did not ligate blunt ends of linear form which were unavoidably generated during the preparation of the nicked circular dsDNA.

**Figure 4.**
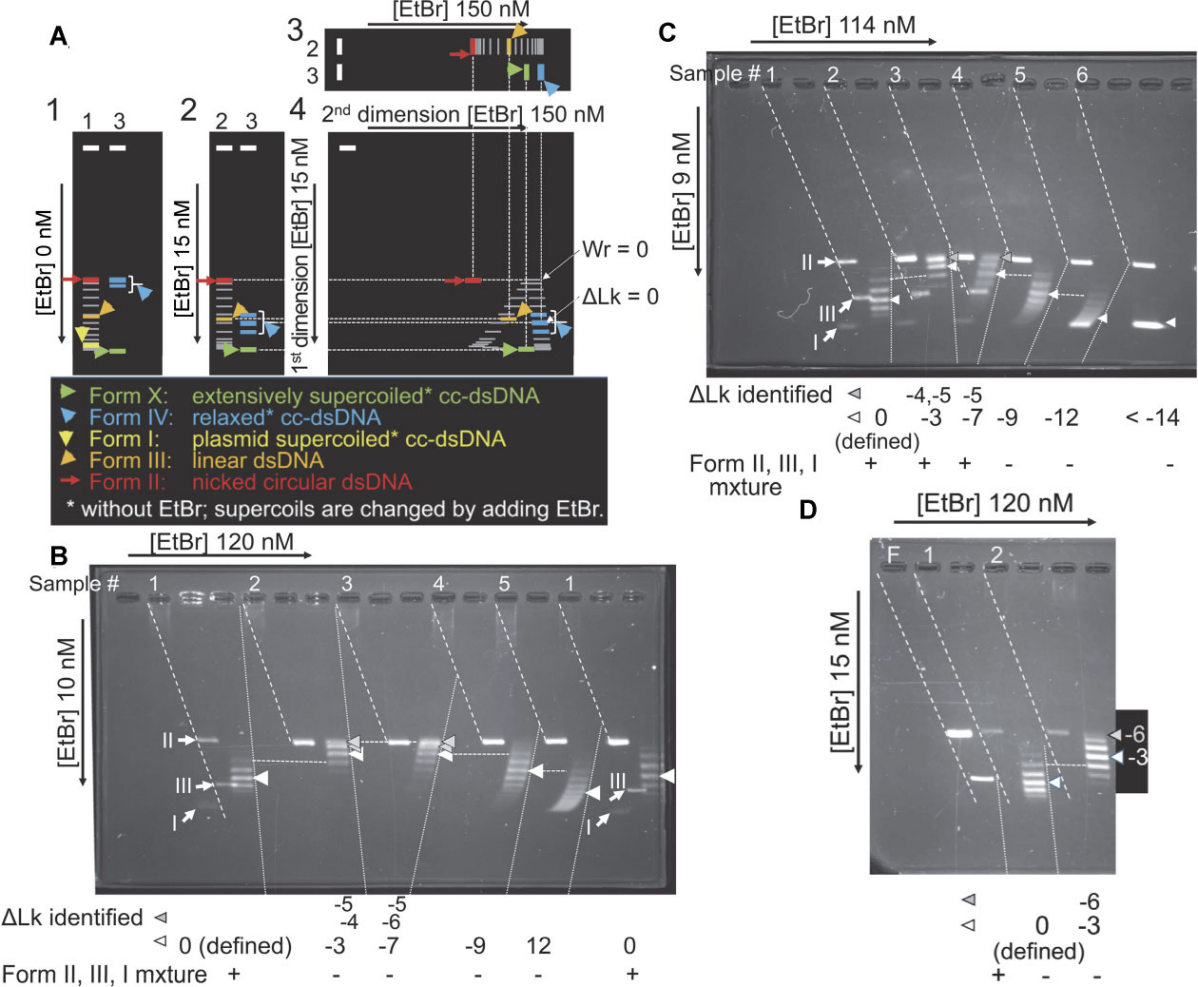
Sensitive assay for dsDNA unwinding. (**A**) Conceptual diagram of two-dimensional agarose-gel electrophoresis to analyze dsDNA unwinding. Definitions of dsDNA forms: Form IV = cc-dsDNA formed by ligation of nicked circular dsDNA or by topoisomerase (nicking-closing enzyme) treatment of cc-dsDNA, in the absence of dsDNA unwinding reagents. Form I = plasmid cc-dsDNA, which has natural negative (right-handed) supercoils in the absence of ethidium bromide. Form X = extensively supercoiled cc-dsDNA, in the absence of dsDNA unwinding reagents, that is formed by ligation of nicked circular dsDNA extensively unwound. Form II = nicked circular dsDNA. Form III = linear dsDNA. Panels 1, 2 and 3: One dimensional electrophoresis in the absence or presence of ethidium bromide. Ethidium bromide concentrations ([EtBr]) are indicated beside each panel. dsDNA samples applied to each sample well (white rectangles) are as follows: Sample 1, mixture of Form I, Form II, Form III, and cc-dsDNA molecules with various linking numbers (topoisomers); Sample 2, mixture of Form II, Form III, and various topoisomers; Sample 3, mixture of Form IV and Form X. Signal pointers are indicated under the set of panels. Panel 4, To determineΔLk (linking difference: the reduction in the linking number from that of Form IV), we analyzed cc-dsDNA samples using two-dimensional electrophoresis. The mixture of Form IV, Form X, and various topoisomers along with Form II and Form III was applied to the sample well (a single white rectangle). The first dimensional run in the presence of ethidium bromide is shown in Panel 2. The second dimensional run in the presence of another concentration of ethidium bromide is shown in Panel 3. In the two-dimensional run, mixture of topoisomers, where Lk is smaller than Lk_0_ (for Form IV), forms an arch of discrete signals, as shown in this panel and in (B–D). cc-dsDNA of which supercoils are completely relaxed (Wr = 0) by the ethidium bromide in the gel migrates to the same position as Form II. Lk_0_ is represented as central prominent band of the band group of Form IV. See Supplementary Data for additional information for topological parameters, two-dimensional electrophoretic analysis, and Determination of ΔLk of each DNA topoisomer. (**B**–**D**) Two-dimensional gel electrophoresis is able to determine linking differences (ΔLk) from 0 to −12, under present conditions: pBlueScript II SK(−) cc-dsDNA SK(−) was treated with calf thymus DNA topoisomerase I (nicking-closing enzyme) in the absence or presence of various concentrations of ethidium bromide at 37°C. Ethidium concentration (μM) used for topoisomerase treatment and topoisomerase amount (Unit/μl) are: Sample 1, 0 μM and 0.2 Unit/μl (to produce Form IV); Sample 2, 0.45 μM and 0.05 Unit/μl; Sample 3, 1.5 μM and 0.05 Unit/μl; Sample 4, 2.3 μM and 0.05 Unit/μl; Sample 5, 2.3 μM and 0.25 Unit/μl; Sample 6, 6.1 μM and 0.05 Unit/μl. After proteins were removed, the treated cc-dsDNA was analyzed by two-dimensional electrophoresis in the presence of ethidium-bromide under three different pairs of concentrations: ethidium bromide concentrations in the first dimension and the second dimensions are 10 and 120 nM, respectively in (B), 9 and 114 nM, respectively, in (C) and 15 and 120 nM, respectively in (D). A mixture of Form I, Form II, and Form III was added to sample 1 in (B), to samples 1–3 in (C), and was electrophoresed as F in (D). Note, the difference of ethidium concentration in the second dimensional run do not influence the determination of ΔLk. The number above each sample well indicates the sample number loaded to the well. Signals of Form I, Form II, Form III, and signal X are shown as I, II, III and X, respectively. The slanting broken lines indicate on which sample well the Form II signal and Form III signal from each sample are aligned. The dotted lines separate signals from each sample (also in Figures 5 and 6). ΔLk for each DNA topoisomer in Samples 2 to 6 was determined (as described in Supplementary Data), defining the central and the most prominent band in sample 1 represents Lk_0_. Horizontal broken lines indicate the bands with the same ΔLks between adjacent samples. White arrowheads indicate the central topoisomer (band) of each sample, and gray ones indicate the topoisomers of which Wr (Writhing number, number of supercoils: see Supplementary Data) is close to 0. ΔLk for each of these topoisomers is shown below the group of bands in each sample. Note that the signals of the topoisomers of which Wr is close to 0 or large are poorly separated (82).

**Figure 5.**
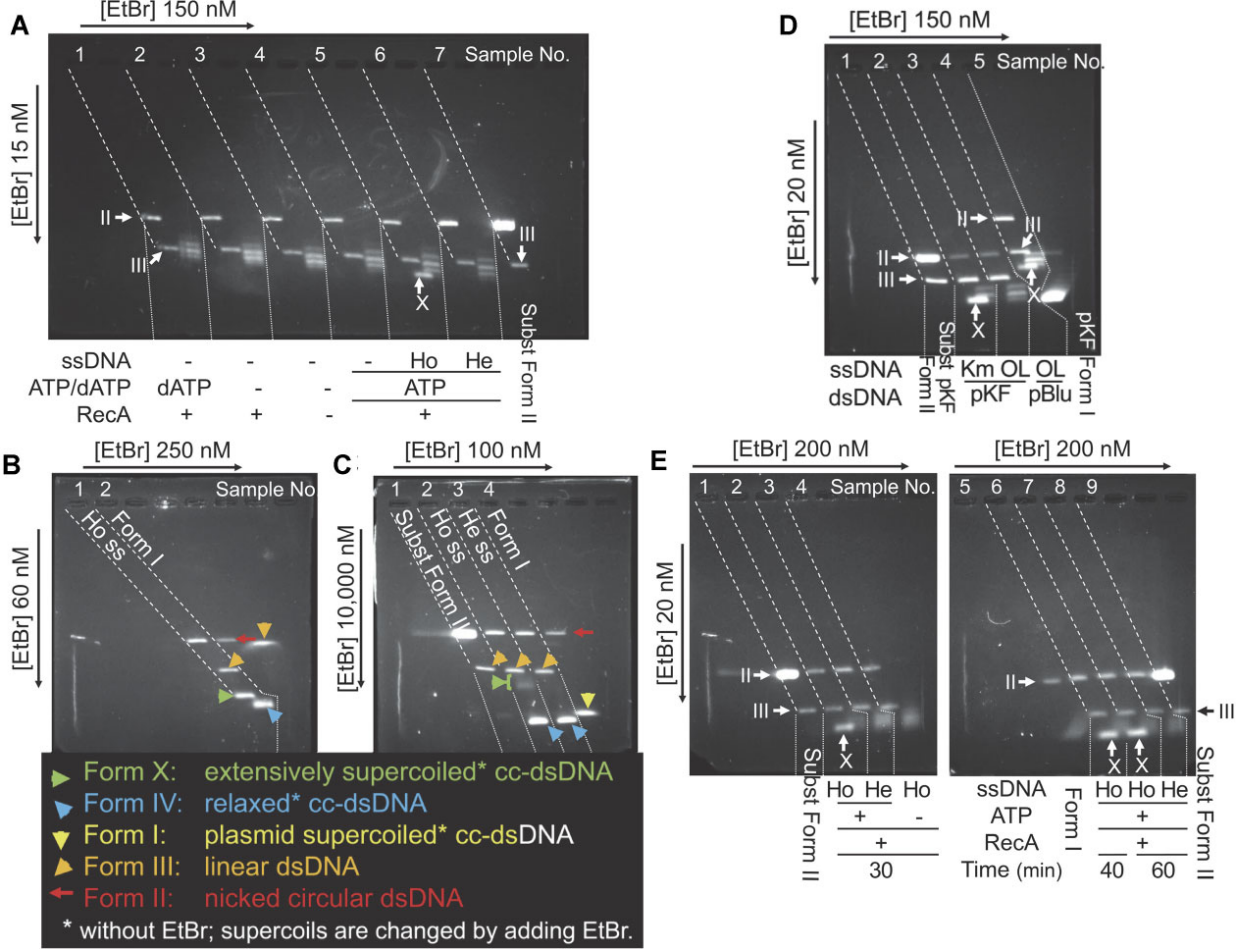
Analysis of unwinding of dsDNA by RecA^+^ at single-turn resolution. (**A**) Unwinding of dsDNA more than a single turn by RecA in the presence of ATP depends on the presence of homologous ssDNA. OL2 (homologous) or Km90 (heterologous) 90-mer ssDNA (1.1 μM in nucleotides, 12 nM in molecules) was incubated with RecA^+^ (4.6 μM) in the presence of ATP (4 mM) for 5 min at 37°C. The unwinding reaction was initiated by the addition of pBlueScript II SK(−) (2961 bp) nicked circular dsDNA (substrate Form II, 10 μM in nucleotides, 1.7 nM in molecules) and continued for 40 min at 37°C. After incubation, T4 DNA ligase was added, followed by incubation for 2 min at 37°C. After the proteins were removed, the DNA samples were analyzed by two-dimensional gel electrophoresis in the presence of ethidium-bromide (15 nM in the first dimension and 150 nM in the second dimension). Ho, homologous; He, heterologous; +, added; −, omitted; Subst Form II, untreated substrate Form II DNA. (**B** and **C**) Signal X is derived from Form X, an extensively supercoiled form of cc-dsDNA. DNA samples were prepared as described in (A) and separated by two-dimensional electrophoresis, in which the ethidium concentrations in the first and second dimensional runs were increased as follows: first dimension, 60 nM and second dimension, 250 nM in (B); first dimension, 10 000 nM and second dimension 100 nM in (C). Ho ss, with homologous ssDNA; He ss, with heterologous ssDNA; Form I, untreated Form I DNA; Broken and dotted lines, see Figure 4B–D. (**D**) Unwinding of dsDNA more than a single turn by RecA solely depends on homologous combination of dsDNA and ssDNA. OL2 or Km90 90-mer ssDNA (1.1 μM in nucleotides, 12 nM in molecules) and pBlueScript II SK(−) or pKF18 (2203 base pairs) nicked circular dsDNA at 10 μM in nucleotides; 1.7 nM (pBlueScript II SK(−)) or 2.3 nM (pKF18) in molecules, were incubated with RecA^+^ in the presence of ATP at 37°C. The ethidium-bromide concentration in the first dimension was 20 nM. Other conditions were the same as those in C. Note that the pKF18 nicked circular dsDNA preparation (substrate Form II) contained more Form III than did pBlueScript II SK(−) substrate Form II, because of the variation in the extent of cleavage by *Alu*I during the nicked circular dsDNA preparation. Km, Km90; OL, OL2; pKF, pKF18; pBlu, pBlueScript II SK(−). (**E**) Homologous ssDNA-dependent extensive unwinding by RecA continues for at least 60 min of incubation and is dependent upon the presence of ATP. DNA samples were prepared as described in (A), with the exception of incubation time, and separated by two-dimensional electrophoresis, in which the ethidium concentrations were 20 nM in the first dimension and 200 nM in the second dimension. Ho, homologous; He, heterologous; +, added; –, omitted. In this series of analyses, Form IV signals were not clearly separated.

**Figure 6.**
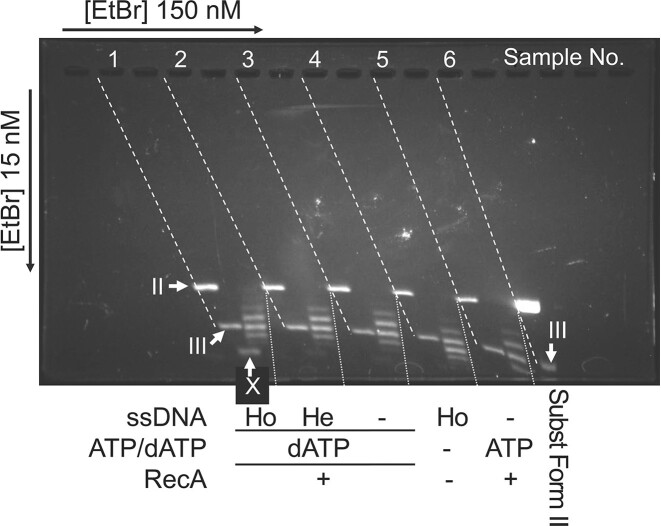
Unwinding of dsDNA by RecA^+^ when ATP is replaced by dATP. The conditions for the reaction were the same as those in Figure 5A, except that ATP was replaced by dATP. Ho, homologous; He, heterologous; +, added, –, omitted. Subst Form II, untreated substrate Form II DNA. Broken and dotted lines, see Figure 4B–D.

When nicked circular dsDNA is ligated, the cc-dsDNA thus formed has a unique linking number (Lk), an integer of the right-handed interwinding of two circular DNA strands (see Supplementary Data for the explanation of the linking number and other topological parameters and details of the topological assay, and also see refs. 65,66). When cc-dsDNA is formed in the absence of double helix unwinding agents such as ethidium bromide or RecA (Form IV DNA), the linking number of Form IV (Lk_0_) is equal to the number of double-helical turns (twisting number, Tw) of the B-form DNA (Tw_0_ = *N*/10, where *N* is the number of base pairs of the dsDNA). Because of fluctuations in the twisting number (not an integer) caused by thermal rotational movement around the axis of the double helix, the linking numbers of a cc-dsDNA population are distributed around a central (≅ average) number. Since the linking numbers are integral, the cc-dsDNA signals (bands) are always a set of discrete bands in gel electrophoretic profiles (see Figure 4), a pair of adjacent bands within a set of signals shows the difference in the linking number by +1 or –1, and the strength of the bands in the set reflects this distribution, as shown in Figures 4B–D; 5A, D, E; 6. If the dsDNA is unwound by *n* turns before ligation, the cc-dsDNA population formed by the ligation has a unique central linking number smaller by *n* than the central Lk (Lk_0_) of the cc-dsDNA population of Form IV (Figure 4B–D). This difference is called the linking difference (ΔLk = Lk – Lk_0_) (67). Two-dimensional electrophoresis under the present conditions separates the signals of the cc-dsDNA molecules with a wide variety of linking numbers smaller than those of Form IV (Figure 4; see Supplementary Data for details), and the numbers of unwinding turns are directly shown as negative ΔLk, the step numbers of upward shift until ΔLk = *ca*. −6 then of shift towards the lower left of the band set in comparison with the central band of Form IV (Figure 4B–D) (or precisely central Lk of negative control cc-dsDNA, Lk_c_; see Supplementary Data); thus, even a single turn of unwinding among even 3000 bp of circular dsDNA at the time of ligation can be detected reliably.

Under the present conditions, the nicked circular dsDNA preparation (substrate Form II) made using the *Alu*I treatment contained a small amount of Form III with blunt ends, formed as a by-product of the *Alu*I treatment, and a small amount of Form II that contained a nick spontaneously formed during storage. Neither the Form III nor the spontaneously formed Form II were ligated under the conditions used in this study. The reaction products after T4 DNA-ligase treatment contained small amounts of suchlike Form II and Form III. The migrations of Form II and Form III are not changed by the presence or absence of ethidium bromide; thus, the sample well, the band of Form II, and that of Form III are aligned on a slanting linear line in two-dimensional gel electrophoretic profiles (broken lines in Figure 4, B - D and the following electrophoretic profiles).

### dsDNA unwinding is detected only after sequence homology with ssDNA is recognized

D-loops are formed when dsDNA and homologous ssDNA are incubated with RecA in the presence of ATP, although D-loop formation with nicked circular dsDNA is slower than that with cc-dsDNA with negative supercoils (46). As shown in Figure 5A, when nicked circular dsDNA was incubated for 40 min with homologous ssDNA and RecA in the presence of ATP, and ligated by T4 DNA ligase (Sample 5), a signal, labeled ‘X’, was observed, in addition to the band set at the positions of those of Form IV (compared with the cc-dsDNA formed in the absence of RecA, Sample 3). The mobility of signal X in the first dimensional run was retarded, compared with that of Form IV, when the ethidium-bromide concentration in the first dimensional run was increased to 60 nM and 10 000 nM (Figure 5B and C, respectively). We chose two sets of ethidium bromide concentrations to show that signal X is that of Form X, by relative mobilities of signal X and Form I. Indeed, when the concentration was 60 nM, signal X (Sample 1) migrated much faster than Form I (Sample 2) in the first dimensional run (Figure 5B), but when the concentration was 10 000 nM, signal X (Sample 2) migrated more slowly than the signal of Form I (Sample 4) and was broader and appeared to split into a set of bands (Sample 2, Figure 5C). This indicates that the DNA product producing the signal X was cc-dsDNA with a much higher number of right-handed (negative) supercoils (Form X) than Form I; thus, the dsDNA producing the signal X was extensively unwound when the nicks of the dsDNA were ligated.

In all the above experiments, we used pBlueScript II SK(−) nicked circular dsDNA as the dsDNA, OL2 (90-mer) as homologous ssDNA, and Km90 (90-mer) as heterologous ssDNA. Km90 is part of the sequence of pKF18 plasmid dsDNA. We then replaced the pBlueScript II SK(−) dsDNA with pKF18 dsDNA. As shown in Figure 5D, Form X was detected only when the combination of dsDNA and ssDNA was pKF18 and Km90 (Sample 2), like the combination of pBlueScript II SK(−) and OL2 (Sample 4; i.e. homologous combination). Form X was not detected when pKF18 dsDNA was incubated with OL2 (Figure 5D, Sample 3), as with pBlueScript II SK(−) dsDNA incubated with Km90 (i.e. heterologous combination; Figure 5A, Sample 6). These observations indicate that the production of Form X, and thus, dsDNA unwinding, is solely dependent on the homologous combination of dsDNA and ssDNA.

The amount of Form X increased during incubation of the nicked circular dsDNA with RecA, homologous ssDNA, and ATP until 40–60 min before the addition of T4 DNA ligase (Figure 5E, Samples 2, 6 and 7). Form X formation is ATP dependent (Figure 5E, Sample 4). We removed an ATP-regenerating system in the unwinding assay, but this result indicates that the supply of ATP to the dsDNA unwinding mediated by RecA is sufficient for at least 60 min, even in the absence of the ATP-regenerating system.

RecA requires ATP to promote D-loop formation and dissociation. ATP can be replaced with dATP (see ref. 32), and dATP could be a natural cofactor of RecA *in vivo* (68,69). We tested dATP for unwinding of dsDNA by RecA. As shown in Figure 6, in the presence of dATP rather than ATP, we again observed that Form X formation was dependent on the presence of homologous ssDNA (Sample 1 versus Sample 2).

Compared with cc-dsDNA (Form IV) formed in the absence of RecA (Sample 3 in Figure 5A; Sample 4 in Figure 6) or in the absence of dATP or ATP (Sample 2 in Figure 5A), a slight upward shift of the 4 bands formed in the presence of RecA and dATP or ATP was observed in the absence of the ssDNA (Sample 1 in Figure 5A; Sample 3 in Figure 6 for dATP, and Sample 4 in Figure 4C and Sample 5 in Figure 6 for ATP). This shift of the band set is less than one helical turn out of 300 turns (pBlueScript II) of the double helices of the dsDNA. This shift is likely to be caused by non-specific RecA interactions with the dsDNA and is not related to D-loop formation, since, as described in the Introduction, the search for homologous sequence for D-loop formation is initiated by the binding of dsDNA to ATP-activated RecA–ssDNA spiral filaments. When the dsDNA was incubated with heterologous ssDNA and RecA in the presence of sufficient amounts of ATP or dATP, search for sequence homology continued until the reaction was terminated. Contrary to the result predicted by the ssDNA annealing after dsDNA-strand separation model, when the homologous ssDNA was replaced by heterologous ssDNA in the presence of either ATP or dATP, we did not detect even a single step shift for the set of the bands of cc-dsDNA formed, and found no additional signals such as X of the cc-dsDNA (Sample 6 in Figure 5A; Sample 2 in Figure 6), compared with the band set of the cc-dsDNA formed in the absence of ssDNA (Sample 4 in Figure 5A; Sample 3 in Figure 6). In addition, the set of bands detected in the products of the reaction containing RecA and dsDNA and homologous ssDNA (Sample 5 in Figure 5A; Sample 1 in Figure 6) represented the cc-dsDNA that failed to detect sequence homology with ssDNA until ligation by T4 DNA ligase. The position of the set of the bands in the gel was exactly the same as that of the set formed in the absence of ssDNA (Sample 4 in Figure 5A; Sample 3 in Figure 6). Thus, the dsDNA was not unwound even a single turn during search for a homologous sequence between the dsDNA and ssDNA in RecA-ssDNA spiral filaments.

Thus, the results obtained by the sensitive unwinding assay show that dsDNA is not unwound even a single turn until sequence homology with ssDNA is recognized in the RecA-ssDNA-dsDNA nucleoprotein complex, in the presence of either ATP or dATP.

## Discussion

Aromatic residues at the tip of β hairpin are implicated in strand separation of dsDNA in DNA helicases and a DNA replisome, and in the cryo-EM structures of the RecA-ATPγS-ssDNA-dsDNA complex, as described in the Introduction. In this study, we found that the conserved aromatic residue at the tip of a β hairpin loop, Phe203 on the L2 loop of RecA, is not essential to homology recognition between dsDNA and ssDNA by RecA; instead, we found that Phe203 is only required for D-loop processing, detected as D-loop dissociation after homology recognition (Figure 3). This dispensability of Phe203 to D-loop formation suggests homology recognition without the strand separation of dsDNA in RecA-mediated D-loop formation. In support of this idea, our sensitive unwinding assay, wherein the search for homologous sequence between dsDNA and ssDNA by RecA continued without completion (i.e. heterologous combination of dsDNA and ssDNA; Figure 3), showed that dsDNA was not unwound even a single turn, and that dsDNA unwinding more than one turn depends on the presence of homologous ssDNA (Figure 5, A and D, and Figure 6).

Although their ability to do so is reduced compared with RecA^+^, F203A and other Ala replacements of amino-acid residues Met197 and Asn205 on the L2 loop are all able to promote D-loop formation (Figure 3 and Supplementary Figure S2). This result shows that these amino-acid residues on the L2 loop are not essential to D-loop formation, but rather play an auxiliary role. This observed reduction may be explained by the loss of optimized interactions between dsDNA or ssDNA and the L2 loop through these amino-acid residues (42,51).

The dispensability of Phe203 in D-loop formation by RecA and its indispensability in D-loop processing indicate that strand separation, by the stacking of Phe203 with the last base pairs of the dsDNA regions, occurs after dsDNA and ssDNA mutually recognize homologous sequence. It is likely that this strand separation establishes a D-loop (42) and initiates D-loop processing (37), even if a nascent product containing a hybrid-duplex is not an authentic D-loop.

Soon after we discovered the RecA-promoted D-loop formation (15,16), we showed that in the presence of ATPγS, RecA unwound dsDNA depending on the presence of heterologous ssDNA; this was viewed as evidence in support of the ssDNA annealing after dsDNA-strand separation model (70,71). ATPγS is a non-hydrolyzable ATP-analogue that stabilizes the binding of RecA to dsDNA and ssDNA. However, we found in a later study that, in the presence of ATP, which is likely a natural cofactor for RecA-mediated D-loop formation and dissociation, ssDNA-dependent dsDNA-unwinding by RecA requires homology between dsDNA and ssDNA (37,72,73). These later studies revealed that the heterologous ssDNA-dependent unwinding of dsDNA by RecA observed in the preceding studies (70,71) was unique to the presence of ATPγS, rather than ATP (37). Note that the cryo-EM structures described in the Introduction were studied using RecA-ssDNA-dsDNA complexes formed in the presence of ATPγS (42).

The homologous ssDNA-dependent dsDNA unwinding by RecA obtained in the presence of ATP described above (37,72) did not exclude the possibility that a small hybrid-duplex core of 6–8 bp is formed after dsDNA-strand separation; indeed, since the nascent hybrid-duplex product had been estimated to be much bigger because of the low resolution of the experiments in those days (100s base pair: 39,74), the analysis was not designed to exclude this possibility, as described below. First, the change in the cc-dsDNA linking number was analyzed by one dimensional electrophoresis, which, because of the overlap of signals, cannot differentiate the small changes induced by an agent from those of cc-dsDNA relaxed in the absence of the agent (i.e. Form IV). Second, the assay was carried out using cc-dsDNA and topoisomerase, thus leaving open the possibility that topoisomerase was inactivated after the RecA reached the termination of the reaction, and during this period, some relaxation of the cc-dsDNA could occur due to the residual topoisomerase activity. Thus, in the present study, in addition to introducing two-dimensional gel electrophoresis, we used nicked circular dsDNA as the dsDNA substrate and ligated all nicked circular dsDNA by T4 DNA ligase in the presence of active RecA with ssDNA and ATP. Since the ligation is irreversible and since no rotational stress is accumulated in the nicked circular dsDNA, even a single turn of unwinding by RecA in dsDNA of 3000 bp is reliably detected.

So the question remained: how is base-sequence homology between ssDNA and dsDNA recognized without dsDNA-strand separation? dsDNA is stable at the so-called physiological temperature, and its strands are separated cooperatively at a unique temperature. However, at the physiological temperature, each base pair opens and pairs repeatedly, and the lives of A–T base pairs and G–C base pairs differ (milliseconds and 10s milliseconds, respectively), indicating that the opening of a base pair is not cooperative but occurs at random (75,76). Under conditions that disrupt base-stacking within dsDNA, such as the local expansion between base pairs by thermal molecular movements or the bending of dsDNA, unpaired bases rotate to be exposed outside of the dsDNA by the interconversion of sugar puckers (48); the exposed bases can form Watson–Crick-type base pairs with bases of ssDNA to search for sequence homology by RecA without strand separation (see ref. 25 for review).

Within living cells, dsDNA recognizes homologous ssDNA and also homologous ssRNA in various biological processes, such as R-loop formation *in trans* in gene-expression regulation (77) and CRISPR-Cas systems, in addition to homologous recombination and telomere-loop formation (see ref. 25 for review). In almost all cases, homologous sequence search for hybrid-duplex formation is explained by ssDNA–ssDNA or ssDNA–ssRNA annealing after dsDNA-strand separation. dsDNA-unwinding associated with dsDNA strand separation causes topological problem that antagonizes the progression of the reaction, i.e. rotational stress within dsDNA. This problem is important when one considers the *in vivo* environment of dsDNA. The search for homologous sequence for hybrid-duplex product-formation with homologous ssDNA or ssRNA without dsDNA-strand separation does not generate stress within the dsDNA; this would be an advantage for dsDNA to express homology-dependent functions quickly and efficiently *in vivo* and also *in vitro*. Our finding of homology recognition without dsDNA-strand separation would be a clue to reveal a previously unrecognized intrinsic chemical function of the dsDNA molecule (see refs. 25,78).

## Supplementary Material

gkad1260_Supplemental_File

## Data Availability

The data underlying this article are available in the article and in its online Supplementary material.
