# Supplementary material for: Homology recognition without double-stranded DNA-strand separation in D-loop formation by RecA
Source: Nucleic Acids Res. 2024 Jan 12;52(5):2565–77. doi: 10.1093/nar/gkad1260 (PMC10954442; doi:10.1093/nar/gkad1260)
Supplement: gkad1260_Supplemental_File [file gkad1260_supplemental_file.pdf]

Supplementary Data

Homology recognition without double-stranded DNA-strand separation in D-loop formation by RecA

Takehiko Shibata [1,2,3]*, Shukuko Ikawa [3], Wakana Iwasaki [4], Hiroyuki Sasanuma [2], Hisao Masai [2] and Kouji Hirota [1]*

[1] Department of Chemistry, Graduate School of Science, Tokyo Metropolitan University

[2] Genome Dynamics Project, Department of Basic Medical Sciences, Tokyo Metropolitan Institute of Medical Science

[3] Cellular & Molecular Biology Laboratory, RIKEN

[4] Laboratory for Translation Structural Biology, RIKEN Center for Biosystems Dynamics Research

Topological parameters.

When nicked circular dsDNA is ligated, the cc-dsDNA thus formed has a unique linking number (Lk), an integer of the right-handed interwinding of two circular DNA strands (see refs. 65,66). A group of cc-dsDNA with the same base sequence but different Lk is called DNA topoisomer. Twisting number (Tw) is the number of double helical turn of the dsDNA. In the absence of reagents that unwind the double helix, the cc-dsDNA formed (Form IV) by the ligation of nicked circular dsDNA or topoisomerase I-treatment has a linking number (Lk_0) that is identical to Tw_0. Tw_0 is Tw of B-form DNA (1 turn/10 base pair), and thus, $LK_0 = Tw_0 = N/10$, where N is total number of base pairs of cc-dsDNA examined. Because of fluctuations in the twisting number (not an integer) caused by thermal rotational movement around the axis of the double helix, the linking numbers of a cc-dsDNA population are distributed around a central (≅ average) number. Lk_0 is represented as the central most prominent band of the set of 3 or 4 bands of Form IV in the electrophoretic profile, under the present experimental conditions (Sample 1 in Fig. 4. B - D, Sample 3 in Fig. 5A, Sample 4 in Fig. 6). If dsDNA is unwound before ligation by *n* turns, the cc-dsDNA formed by the ligation has a unique negative linking difference, ΔLk where $\Delta Lk = Lk - Lk_0$. After ligation, the unwinding reagents are removed, the cc-dsDNA acquires negative (right-handed) supercoil (or negative (left handed) solenoid, which is not considered in this study), of which number is termed, writhing number (Wr). Among the three topological parameters of cc-dsDNA, there is a simple relationship: Lk = Wr + Tw (see refs. 65,66). Experimental conditions, such as the presence of Mg^{2+} and non-specific interactions with proteins, cause a small change in Tw.

Additional information about two-dimensional electrophoresis

In agarose-gel electrophoresis, the migration rate of cc-dsDNA depends on the absolute number of supercoils (Wr) and is independent of handedness: negative (right-handed) or positive (left-handed). The signals (bands) of the cc-dsDNA molecules migrating near to the Form II signal (Form IV in the

absence of ethidium bromide; Fig. 4A panel 1) and also near the topoisomer signals that migrates at maximum rates (the 1st dimensional run of Form I and Form X in Fig. 4A panels 1, 2 and 4, and Fig. 5, A, B, D and E, and Fig. 6; topoisomers of which ΔLk is less than -12 in Figs. 4B and 4C; Form IV in Fig. 5, B and C) are poorly separated and tend to overlap (82). Thus, the small degree of unwinding is not measurable within these regions.

Ethidium bromide intercalates between base pairs of dsDNA and unwinds dsDNA; it thus reduces the number of double-helix turns. If the dsDNA is cc-dsDNA, this reduction in the number of double-helix turns (Tw) results in an increase by the equal number of Wr in the relationship denoted by the equation described above, Lk = Wr + Tw (see refs. 65,66). As the ethidium-bromide concentration increases, the extent of dsDNA unwinding increases (represented by a decrease in Tw), and Form IV acquires a positive Wr to migrate at a higher rate than Form II in gel electrophoresis. At an appropriate concentration (e.g. 15 nM), the signals of Form IV are well separated (Fig. 4A Panel 2; in the first dimensional electrophoresis in Fig. 4, B - D; Fig. 5, A, D – E, Fig. 6), and a small change in the linking number is sensitively detected as a stepwise shift of the band set (in the first dimensional electrophoresis in Fig. 4, B – D). To broadly count ΔLk, the reduction in the linking number from that of Form IV, we analyzed cc-dsDNA samples using two-dimensional electrophoresis, as shown in Fig. 4A Panel 4. In two-dimensional electrophoresis in the presence of ethidium bromide at 9-15 nM and 110-150 nM in the first and second dimensions, respectively, cc-dsDNA molecules, with various ΔLk, form an arch of discrete signals, as shown in Fig. 4A panel 4.

Determination of ΔLk of each DNA topoisomer

ΔLk for each DNA topoisomer was determined by counting the number of bands starting from the central most prominent band of Form IV (Reference band shown in Sample 1 in Fig. 4, B-D) to the band of the topoisomer in electrophoretic profiles. Upward steps of bands to the top followed by towards the lower left steps indicates a decrease in ΔLk (-1, -2, -3 and so on; see Fig. 4A, panel 4). Sometimes, when their Wr is close to 0, a pair of topoisomers with adjacent ΔLk numbers is indistinguishable, because they migrate at almost the same rate as Form II in the first dimension. The overlap of signals was identified by comparison of electrophoretic profiles in the presence of different ethidium bromide conditions in the first dimension (Fig. 4, B - D) and by the brightness of the signal. For example, the bands of which ΔLk is -5 and -6 overlap partly in Fig. 4B, but are separated in Fig. 4C and Fig. 4D. The bands of which ΔLk is -4 and -5 overlap completely in Fig. 4C, but are separated in Fig. 4D. The ΔLk of the band of which Wr is 0, is -5 in Fig. 4B and overlapped -4 and -5 in Fig. 4C, and -6 in Fig. 4D.

The turn number of the double helix-unwinding just before the ligation (or during topoisomerase I-treatment) by a specified interaction is determined as described in the main text, but more precisely determined by $\Delta Lk_{ave} - \Delta Lk_c$, where ΔLk_{ave} is the average ΔLk of a set of bands of cc-dsDNA obtained under the specified conditions and ΔLk_c is the average of ΔLk of the set of bands of cc-dsDNA obtained from the negative control. In this study, ΔLk_c that was resulted from incubation with RecA and

ATP or dATP, but without ssDNA, was less than I.

It should be noted that the migration of cc-dsDNA topoisomers and that of Form III are determined by different factors in addition to their size: topoisomers are influenced by their supercoils which is sensitive to the environments, but Form III is not. Thus, in electrophoretic profiles, Form III is used as an internal marker but is not accurate one: the relative positions of Form III and bands of topoisomers are variable.

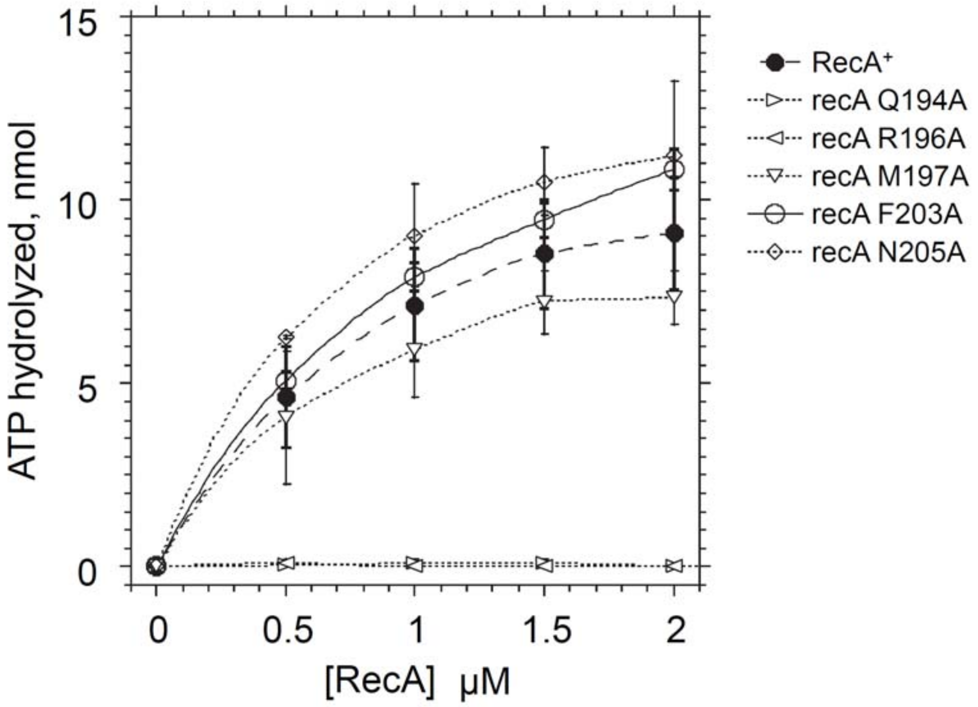

Supplementary Figure 1. ssDNA-dependent ATPase activities of RecA variants.

[α-^{32}P]ATP (26 nmol at 1.3 mM in 20 µL reaction mixture) was incubated with M13 phage ssDNA (10 µM) and various RecA variants at the indicated amounts for 30 min at 37°C. After the reaction was terminated, the amount of ADP formed during the incubation was measured.

RecA^{+}, N=8-10; for other RecA variants, N = 2 - 6.

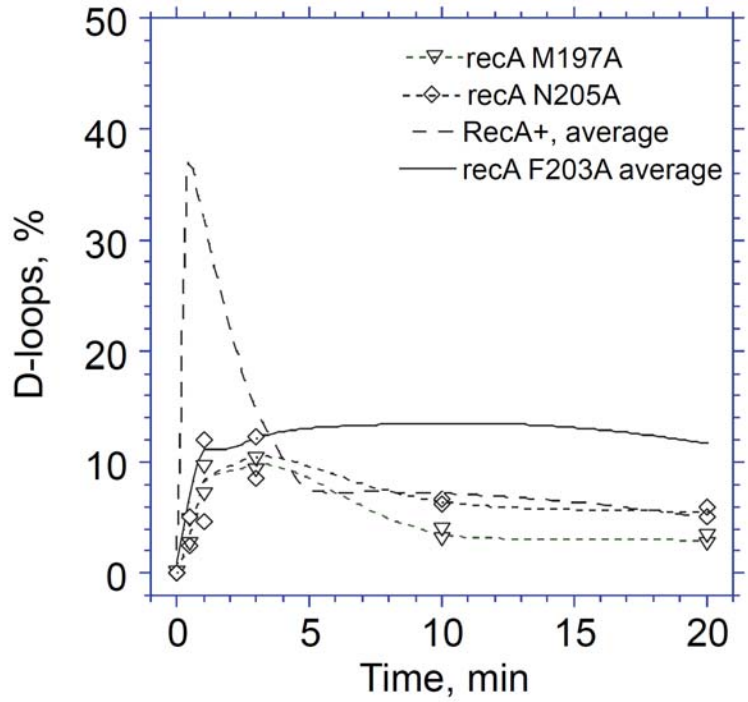

Supplementary Figure 2. D-loop formation and subsequent D-loop dissociation by RecA with Ala replacements of DNA-interacting amino-acid residues on the L2 loop.

The experimental conditions were described in Fig. 3. All data obtained were plotted. Broken lines and solid lines without symbols represent $RecA^{+}$ and recA F203A, respectively, copied from Fig. 3B.

RecA M197A and RecA N205A formed D-loops at a similar rate as recA F203A, but like $RecA^{+}$, dissociated the D-loops once formed.
